# Effect of Notch1 on Vocal Fold Re‐Epithelization After Acute Injury

**DOI:** 10.1002/lary.70760

**Published:** 2026-07-15

**Authors:** Jie Cai, Susan L Thibeault, Vlasta Lungova

**Affiliations:** ^1^ Department of Otolaryngology Head and Neck Surgery University of Wisconsin Madison Madison Wisconsin USA

**Keywords:** epithelium, Lrig1, Notch1, vocal fold

## Abstract

**Objective:**

To investigate the role of Notch1 signaling in Lrig1‐expressing vocal fold (VF) epithelial stem cells during epithelial repair following acute injury.

**Methods:**

Lrig1Cre^ERT2/+^, Lrig1Cre^ERT2/+^Notch1^F/+^, and Lrig1Cre^ERT2/+^Notch1^F/F^ mice were generated. Cre‐mediated recombination was induced by tamoxifen (100 mg/kg, intraperitoneally) on two consecutive days. Acute epithelial injury was induced by naphthalene (NAPH; 250 mg/kg, intraperitoneally), while corn oil was used as a control. Whole larynges were collected at 1, 3, and 7 days post‐injury for histological and immunofluorescence analyses.

**Results:**

Following injury, conditional deletion of Notch1 in Lrig1‐expressing epithelial stem cells disrupted epithelial repair, with the most pronounced phenotype in homozygous Lrig1Cre^ERT2/+^Notch1^F/F^ mice. Expansion of P63 positive basal cells and increased Ki‐67 positive proliferating cells peaked at 3 days and persisted through 1 week post injury. Moreover, epithelial stratification was altered, as indicated by expansion of KRT14 positive epithelial layers and increased expression of the differentiation markers KRT13 and KRT17. Notably, accumulation of IBA1 positive immune cells increased in VF mucosa at 3 days and remained elevated at 1 week, indicating prolonged inflammation.

**Conclusion:**

Collectively, these results suggest that Notch1 plays an important role in regulating epithelial cell fate and coordinating the timing of repair processes in the VF epithelium following acute injury.

**Level of Evidence:**

N/A.

## Graphical abstract

Conditional deletion of Notch1 in Lrig1‐expressing vocal fold epithelial cells impaired re‐epithelialization following naphthalene injury, resulting in sustained basal cell proliferation, abnormal epithelial differentiation, and prolonged inflammatory cell infiltration. These findings demonstrate that Notch1 signaling is essential for coordinating vocal fold epithelial regeneration and immune responses after injury.

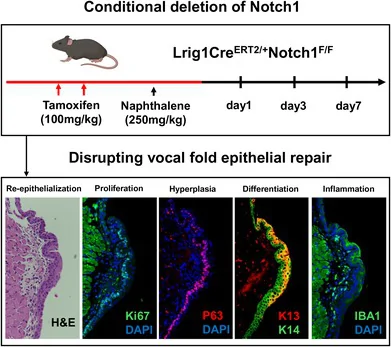

## 1 Introduction

Vocal folds (VF) are multilayered structures composed of epithelium, lamina propria, and muscle [1]. The VF epithelium serves as a physical barrier that protects underlying tissue from mechanical and chemical injury [2]. VF are lined with the stratified squamous epithelium consisting of a basal layer and suprabasal layers [3]. Various insults, including surgical intervention, environmental and systemic irritation, and vibratory injury, can disrupt epithelial integrity and compromise barrier function [4, 5, 6, 7]. Following injury, the epithelium undergoes a repair process that involves activation of a normally quiescent population of epithelial stem cells—typically basal and/or immature secretory cells that undergo transdifferentiation [8]‐followed by proliferation and subsequent differentiation to restore tissue [3, 4, 9]. The process of epithelial regeneration needs to be precisely coordinated to prevent abnormal remodeling and impaired tissue function.

Notch signaling is one pathway that regulates epithelial homeostasis and regeneration, particularly in progenitor and stem cell populations. In the airway epithelium, Notch signaling regulates epithelial cell fate by promoting the formation of parabasal cells, which subsequently differentiate into secretory or ciliated cells [10, 11]. Suppression of Notch signal impairs the luminal differentiation in KRT5^+^ basal stem cells, leading to basal cell accumulation [12]. In the esophagus, Notch governs squamous differentiation, participates in epithelial development, and is required for the specification of esophageal progenitor cells [13, 14, 15]. In the same tissue, conditional deletion of Notch in Lgr5^+^ cells increased goblet‐like cell differentiation, reduced crypt fission, and decreased tumor formation [16]. Recently, we showed that, in the larynx and VF, Notch is essential for maintaining quiescence in Lrig1 (leucine‐rich repeats and immunoglobulin‐like domains 1) epithelial stem cells. Disruption of Notch1 signaling during homeostasis compromises their quiescent state [17], leading to epithelial hyperplasia, expansion of secretory cell populations, and excessive mucus production [17]. However, it remains unclear how Notch1 depletion influences cell fate decisions in Lrig1^+^ cells during VF epithelial repair.

This study investigated the role of Notch1 during VF re‐epithelialization following naphthalene (NAPH) injury, using Lrig1Cre^ERT2^ mice to target tissue‐resident stem cells in laryngeal and VF mucosa [17, 18]. To induce VF epithelial injury, we employed NAPH, a polycyclic aromatic hydrocarbon present in tobacco smoke that is widely used to model acute airway epithelial injury in mice [19, 20]. We hypothesized that deletion of Notch1 in Lrig1‐expressing cells would disrupt their proliferation, differentiation, and the timing of VF epithelial repair, resulting in impaired epithelial regeneration. Furthermore, defective repair may be accompanied by enhanced immune cell infiltration into the injured mucosa and epithelial hyperkeratinization, both of which could compromise VF vibratory function and promote persistent inflammation. Taken together, this study provides new insight into the molecular mechanisms underlying VF mucosa healing, emphasizing the importance of Notch1 signaling in regulating epithelial regeneration and immune responses following NAPH injury.

## 2 Methods and Materials

### 2.1 Mouse Models and Tissue Harvest

Lrig1‐Cre^ERT2^ mice (IMSR_JAX:018418) and Notch1flox mice (IMSR_JAX:006951) were obtained from The Jackson Laboratory. The following genotypes were used in this study: Lrig1‐Cre^ERT2^, Lrig1Cre^ERT2/+^Notch1^F/+^, and Lrig1Cre^ERT2/+^Notch1^F/F^. To induce Cre‐mediated recombination, tamoxifen (Millipore Sigma, Cat. No. T5648‐1G) was administered intraperitoneally on two consecutive days. In order to increase the survival of mice, we reduced the dosage of tamoxifen to 100 mg/kg, comparing with the previous study [17]. Lrig1Cre^ERT2/+^ females were crossed with Notch1^F/+^ males to generate Lrig1Cre^ERT2/+^Notch1^F/+^ heterozygotes. Lrig1Cre^ERT2/+^Notch1^F/+^ were crossed to Notch1^F/F^ to generate conditional homozygous mutants Lrig1Cre^ERT2/+^Notch1^F/F^. Mouse genotypes were confirmed by PCR analysis of genomic DNA isolated from ear punch tissues. Detailed genotyping methods are provided in the Supporting Informations, and representative results are shown in Figure S1. Primer sequences used for genotyping are listed in Table S1. All mice were housed in the University of Wisconsin‐Madison Biomedical Research Model Services Laboratory. All animal procedures were approved by the University of Wisconsin‐Madison Institutional Animal Care and Use Committee.

The exact number of mice analyzed varied among staining experiments. H&E staining included *n* = 5 mice per genotype per treatment group and time point. Immunofluorescence analysis included *n* = 4–5 mice per genotype, depending on the marker and time point, with exact sample sizes stated in the corresponding figure legends. In all cases, n represents the number of mice.

### 2.2 Naphthalene Treatment

VF epithelium injury was induced by intraperitoneal injection of NAPH 250 mg/kg (Millipore Sigma, Cat. No. 1275‐1G). Corn oil (Millipore Sigma, Cat. No. C8267) was used as a control. VF tissues were collected at 1, 3, 7 days post‐injury.

### 2.3 Histology and Immunofluorescence

Mouse larynges were fixed in 4% paraformaldehyde, processed, embedded in paraffin, and sectioned at a thickness of 5 μm. Hematoxylin and eosin (H&E) and immunofluorescence staining (IF) were performed as previously described [21]. All antibody information is listed in Table S2. Fluorescence images were acquired using a Nikon Eclipse Ti2 inverted microscope equipped with a DS‐Ri2 camera and NIS‐Elements software.

### 2.4 Quantification of Immunofluorescent Signals

For quantification, the region of interest (ROI) was defined as the entire VF epithelial layer visible in each section to minimize regional sampling bias. The epithelial ROI was manually outlined based on DAPI staining and tissue morphology. Within this ROI, total DAPI^+^ epithelial cells, as well as Ki‐67^+^ and P63^+^ cells, were manually counted, and positive cells were expressed as a percentage of total DAPI^+^ epithelial cells. KRT13^+^ and KRT17^+^ expression was quantified as the percentage of positive immunofluorescent signal area relative to the total epithelial ROI area. The area of positive staining was determined using a predefined threshold, as previously described [22]. All procedures were performed using ImageJ. Measurements were normalized to the total VF epithelial area, and at least three independent biological replicates were analyzed.

### 2.5 Statistical Analysis

Statistical analyses were performed using GraphPad Prism (version 10.0.3). Relative gene expression among multiple groups was compared using one‐way analysis of variance (ANOVA). *p ≤ 0.05 was considered statistically significant.

## 3 Result

### 3.1 Conditional Deletion of Notch1 in Lrig1Cre^ERT2^ Mice Disrupts VF Epithelial Regeneration After Injury

The procedure we used to induce Cre recombination and epithelial injury in our experimental mouse models is illustrated in Figure 1A. At 24 h post‐injury, compared with controls, NAPH injury resulted in a disrupted and thinned epithelium, accompanied by accumulation of cellular debris in the lumen (Figure 1B–F). At 3 days post‐injury, VF epithelium displayed marked epithelial hyperplasia (Figure 1G–K) in all NAPH‐treated groups compared with their corresponding corn oil‐treated controls. This hyperplasia responses were most prominent in Lrig1Cre^ERT2/+^Notch1^F/F^ homozygous mice (Figure 1K) with milder effect in heterozygotes (Figure 1J) and minimal changes in Lrig1Cre^ERT2/+^ mice (Figure 1H). Similar findings were observed at 1 week post‐injury (Figure 1M,O,P) suggesting that loss of Notch1 may disrupt VF epithelial repair.

**FIGURE 1:**
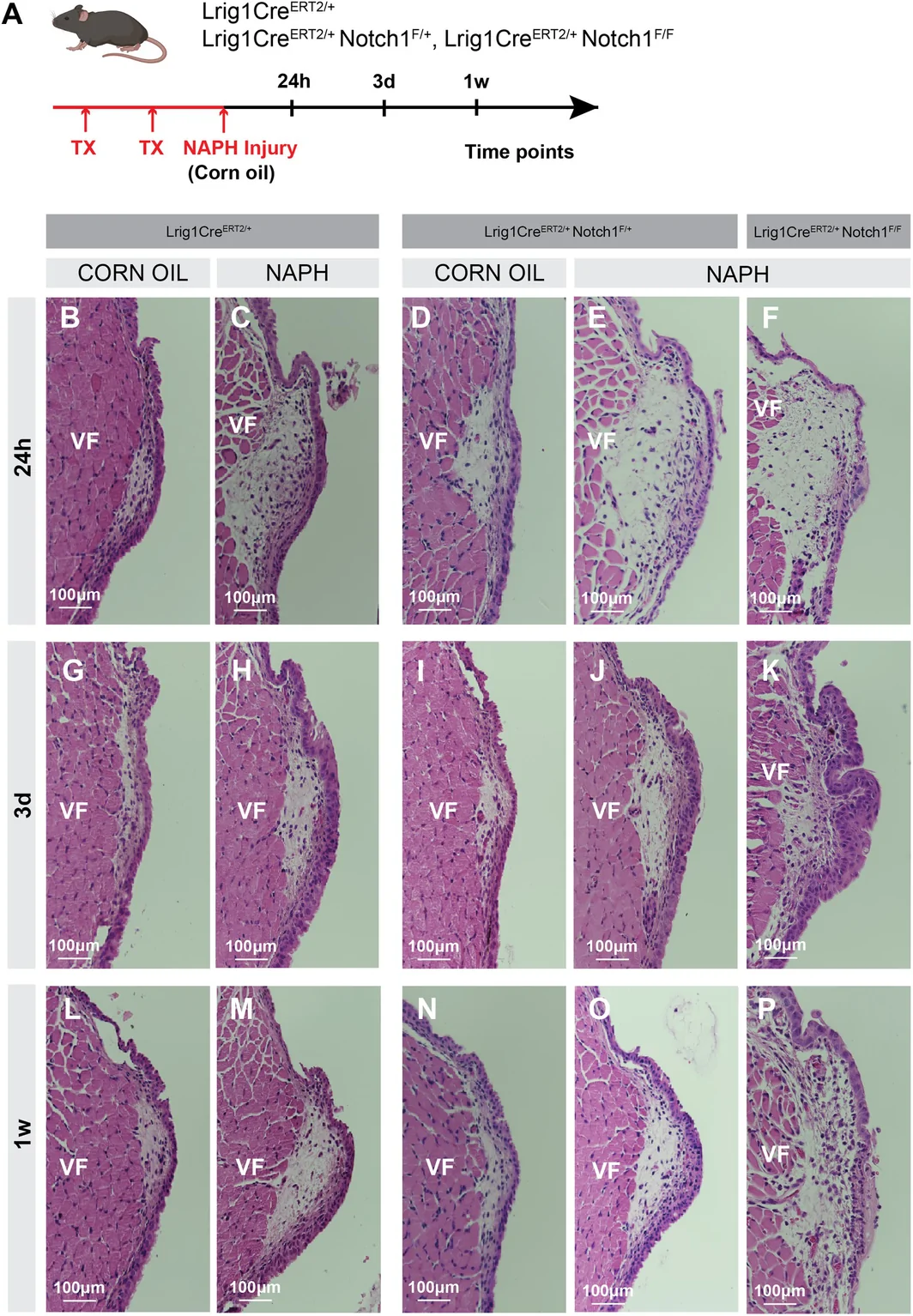
Effect of Notch1 deletion on VF re‐epithelialization following NAPH induced injury in Lrig1Cre^ERT2/+^, Lrig1Cre^ERT2/+^Notch1^F/+^, and Lrig1Cre^ERT2/+^Notch1^F/F^ mice. (A) Schematic illustration of experimental design and strategy. (B–P) Representative H&E staining showing epithelial morphology in VF in corn oil‐treated control and NAPH‐treated mice including at 24 h (B–F), at 3 days (G–K), and at 1‐week post‐injury (L–P). *n* = 5 mice per genotype per treatment group. d, days; h, hours; *n*, number of mice; NAPH, naphthalene; VF, vocal fold; w, week. [Color figure can be viewed in the online issue, which is available at www.laryngoscope.com]

### 3.2 Notch1 Deficiency in Lrig1Cre^ERT2^ ^/+^ Mice Promotes Basal Cell Proliferation and Epithelial Hyperplasia After Injury

Since the morphological changes were most pronounced in Notch1 conditional knockout mice, subsequent analyses focused on comparing Lrig1Cre^ERT2/+^Notch1^F/+^ and Lrig1Cre^ERT2/+^Notch1^F/F^ mice only. At 3 days post‐injury, P63‐positive basal cells markedly expanded in injured animals and extended beyond the basal layer to suprabasal positions compared with corn oil treated animals (Figure 2A–F). This expansion correlated with significant rise in Ki‐67 positive proliferating cells, which predominantly co‐localized with P63^+^ basal cells (Figure 2G–L). Notably, Notch1 deletion further intensified these changes (Figure 2E,F,K,L), as Lrig1Cre^ERT2/+^Notch1^F/F^ mice exhibited significant increase in P63^+^ basal cells (*p* = 0.0020) and Ki‐67^+^ cells (*p* = 0.0002) compared with injured Lrig1Cre^ERT2/+^Notch1^F/+^ mice and corn oil controls (Figure 2Y,Z).

**FIGURE 2:**
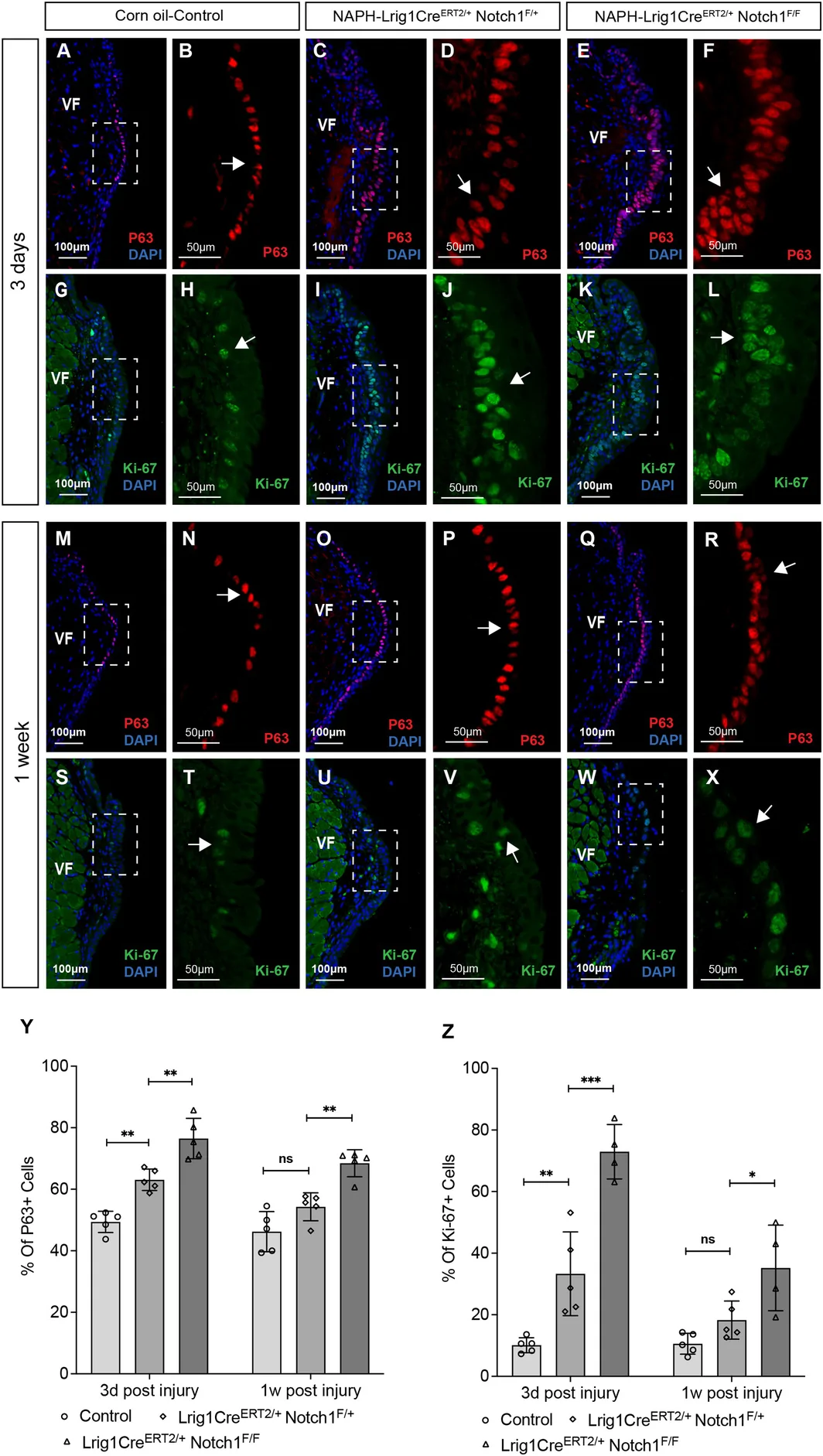
Notch1 deficiency promotes basal cell proliferation and epithelial hyperplasia following NAPH injury in heterozygous (Lrig1Cre^ERT2/+^Notch1^F/+^) and homozygous (Lrig1Cre^ERT2/+^Notch1^F/F^) mice. (A–F, G–L) IF staining for P63 (red) and Ki‐67 (green) in VF epithelium of corn oil–treated controls and NAPH‐injured heterozygous and homozygous mice at 3 days post‐injury. (M–R, S–X) IF staining for P63 (red) and Ki‐67 (green) in VF epithelium of corn oil–treated controls and NAPH‐injured heterozygous and homozygous mice at 1‐week post‐injury. (Y, Z) Quantification of P63‐positive and Ki‐67‐positive epithelial cells in VF epithelium at 3 days and 1‐week post‐injury in corn oil–treated controls and NAPH‐injured heterozygous and homozygous mice. Error bars represent mean ± SD, with individual data points shown. Statistical significance was determined by one‐way ANOVA. *p ≤ 0.05, ns, not significant (*p* > 0.05). For P63 staining, *n* = 5 control, *n* = 5 heterozygote, and *n* = 5 homozygote mice in 3 days and 1 week post injury. For Ki‐67 staining, *n* = 5 control, *n* = 5 heterozygote, and *n* = 4 homozygote mice in 3 days and 1 week post injury. d, days; *n*, number of mice; NAPH, naphthalene; VF, vocal fold; w, week. [Color figure can be viewed in the online issue, which is available at www.laryngoscope.com]

At 1 week post‐injury, epithelial hyperplasia was partially resolved in Lrig1Cre^ERT2/+^Notch1^F/+^ mice, as the P63^+^ basal cells showed only modest and non‐significant increases compared with controls (*p* = 0.0742) (Figure 2M–P), and Ki‐67 positive cells were not significantly different (*p* = 0.3537) (Figure 2S–V). In contrast, Lrig1Cre^ERT2/+^Notch1^F/F^ mice continued to exhibit a significant increase in P63 positive basal cells compared with both controls (*p* < 0.0001) and injured Lrig1Cre^ERT2/+^Notch1^F/+^ mice (*p* = 0.0028) (Figure 2Q,R,Y). Similarly, Ki‐67 positive cells remained significantly elevated compared with controls (*p* = 0.0030) and injured Lrig1Cre^ERT2/+^Notch1^F/+^ mice (*p* = 0.00299) (Figure 2W,X,Z). However, epithelial thickening and basal cell expansion were less pronounced than at the 3 day time point, suggesting partial resolution of injury response.

### 3.3 Conditional Deletion of Notch1 in Lrig1Cre^ERT2^ ^/+^ Mice Disrupts Epithelial Differentiation During VF Repair

At 3 days post‐injury, strong KRT14 positive epithelial layers expanded and spread toward the VF inferior margin into the transitional zone between stratified squamous and respiratory epithelium. Simultaneously, strong KRT13 expression was detected in suprabasal layers of the VF epithelium in both injured heterozygotes and homozygotes but not in corn oil controls (Figure 3A1–C4). Quantitative analysis confirmed a significant increase in KRT13 expression after injury, with the greatest elevation observed in injured Lrig1Cre^ERT2/+^Notch1^F/F^ (*p* = 0.0391) (Figure 3M). At 1 week post‐injury, although epithelial architecture showed partial recovery, KRT13 expression remained elevated in both injured mutant groups (Figure 3D1–F4). Quantitative analyses further showed that KRT13 expression remained significantly higher in injured Lrig1Cre^ERT2/+^Notch1^F/F^ mice than injured heterozygotes (*p* = 0.0287) and corn oil controls (*p* = 0.0040) (Figure 3M).

**FIGURE 3:**
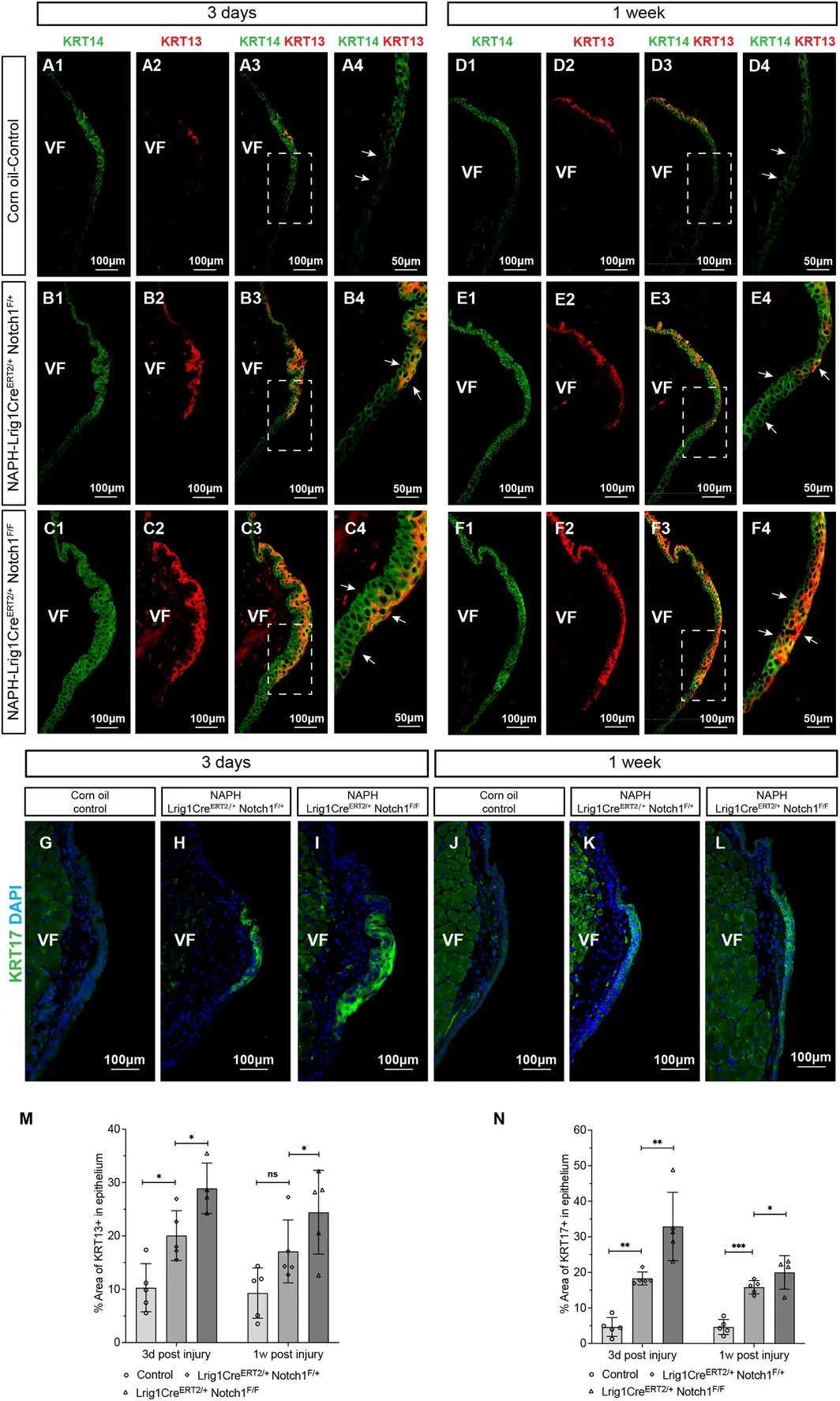
Notch1 deletion in Lrig1+ cell alters epithelial differentiation during VF repair in heterozygous (Lrig1Cre^ERT2/+^Notch1^F/+^) and homozygous (Lrig1Cre^ERT2/+^Notch1^F/F^) mice. (A1–F4) IF showing KRT13 (red) and KRT14 (green) expression in VF epithelium of corn oil–treated controls and NAPH‐injured heterozygous and homozygous mice at 3 days and 1‐week post‐injury. (G–L) KRT17 (green) expression in VF epithelium of corn oil–treated controls and NAPH‐injured heterozygous and homozygous at 3 days and 1‐week post‐injury. (M, N) Quantification of KRT13 and KRT17 positive epithelial cells in controls, NAPH‐injured heterozygous and homozygous mutants. Data are presented as mean ± SD with individual data points. Statistical significance was determined using one‐way ANOVA. * *p* ≤ 0.05, ns, not significant (*p* > 0.05). For KRT13 staining, *n* = 5 control, *n* = 5 heterozygote, and *n* = 4 homozygote mice at 3 days; *n* = 5 control, *n* = 5 heterozygote, and *n* = 5 homozygote mice at 1 week. For KRT17 staining, *n* = 5 control, *n* = 5 heterozygote, and *n* = 5 homozygote mice at 3 days and 1 week post injury. *n*, number of mice; NAPH, naphthalene; VF, vocal fold. [Color figure can be viewed in the online issue, which is available at www.laryngoscope.com]

To further evaluate epithelial differentiation status and aberrant cytokeratin expression, we examined the stress‐associated cytokeratin KRT17 [23]. KRT17 expression was minimal in control VF epithelium (Figure 3G). In contrast, robust KRT17 expression was detected at 3 days following NAPH injury in both heterozygotes and homozygotes (Figure 3H,I). Quantitative analysis further confirmed significantly higher KRT17 expression in the VF epithelium of injured Lrig1Cre^ERT2/+^Notch1^F/F^ mice than injured Lrig1Cre^ERT2/+^Notch1^F/+^ mutants (*p* = 0.0050) (Figure 3N). At 1 week post‐injury, KRT17 expression remained elevated in both mutant groups relative to controls (Figure 3J–L), with a modest but significant increase in homozygous mutants compared with heterozygous (*p* = 0.0309) (Figure 3N), indicating an increased keratin deposition in the restoring epithelium upon Notch1 inactivation.

### 3.4 Conditional Deletion of Notch1 in Lrig1Cre^ERT2^ ^/+^ Mice Enhances Macrophage Accumulation in the VF Mucosa After Injury

At 3 days post‐injury, IBA1 positive cells infiltration in the VF mucosa was substantially higher in both heterozygous and homozygous mutants than in corn oil controls (Figure 4A–F). This increase was significantly greater in Lrig1Cre^ERT2/+^Notch1^F/F^ mice (Figure 4E,F) than in Lrig1Cre^ERT2/+^Notch1^F/+^ mice (Figure 4C,D). At 1 week post injury, Lrig1Cre^ERT2/+^Notch1^F/F^ mice continued to exhibit higher numbers of IBA1 positive cells in VF mucosa than Lrig1Cre^ERT2/+^Notch1^F/+^ mice or corn oil controls (Figure 4G–L), suggesting that loss of Notch1 may promote macrophage accumulation at the injury site, thereby enhancing mucosal inflammation.

**FIGURE 4:**
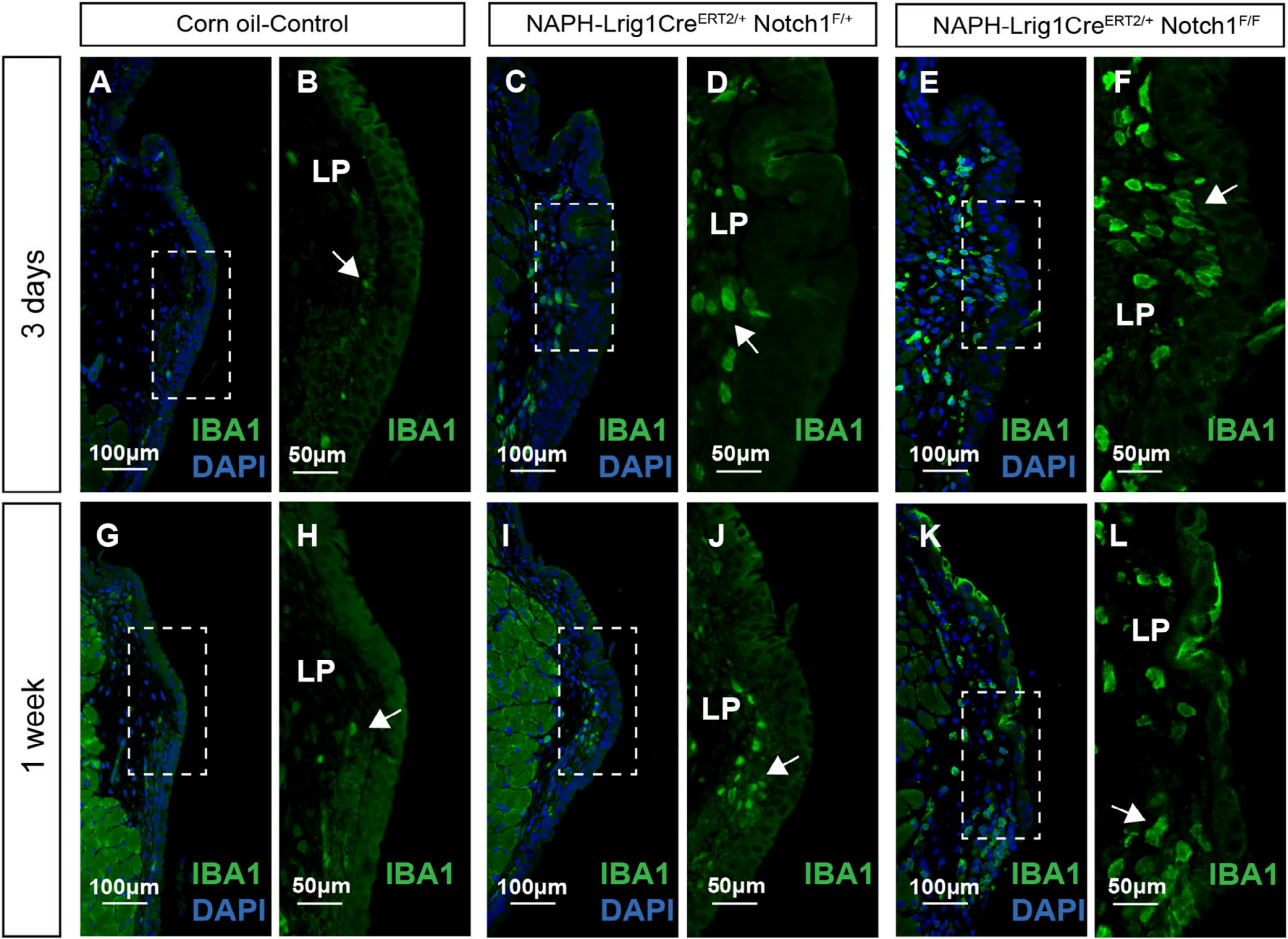
Conditional Notch1 deletion enhances macrophage accumulation in VF mucosa after VF epithelial injury in heterozygous (Lrig1Cre^ERT2/+^Notch1^F/+^) and homozygous (Lrig1Cre^ERT2/+^Notch1^F/F^) mice. (A‐F) IF staining for IBA1 (green) in VF of corn oil–treated controls and NAPH‐injured heterozygous and homozygous mice at 3 days post‐injury. (G‐L) IF staining for IBA1 (green) in VF of corn oil–treated controls and NAPH‐injured heterozygous and homozygous mice at 1‐week post‐injury. For IBA1 staining, *n* = 5 control, *n* = 5 heterozygote, and *n* = 5 homozygote mice at 3 days; *n* = 5 control, *n* = 5 heterozygote, and *n* = 4 homozygote mice at 1 week. d, days; LP, lamina propria, NAPH, naphthalene; *n*, number of mice; w, week. [Color figure can be viewed in the online issue, which is available at www.laryngoscope.com]

## 4 Discussion

In the present study, we demonstrate that conditional inactivation of Notch1 in Lrig1‐expressing cells disrupts VF epithelial regeneration following NAPH‐induced injury, indicating that Notch1 signaling is required to regulate the injury response of these normally quiescent progenitor cells. Notch1 loss in Lrig1^+^ cells led to marked basal cell expansion and abnormal epithelial remodeling, including altered stratification and increased stress keratin deposition. These changes were accompanied by elevated mucosal immune cell infiltration and persisted for up to 1 week post‐injury, particularly in NAPH‐injured Lrig1Cre^ERT2/+^Notch1^F/F^ mice.

Our findings are consistent with previous observations. Quiescent Lrig1 progenitor/stem cells are primarily located among basal cells and immature secretory cells, which can transdifferentiate into basal cells in response to injury [17]. Therefore, basal cell overactivation due to reduced Notch1 signaling during re‐epithelization predominantly affects basal cell proliferation and subsequent differentiation. Moreover, a subset of cells with Lrig1^+^ signal was found in endothelial cells. Endothelial cells serve as key regulators of immune cell infiltration through adhesion molecules and chemokine gradients [24]. Consistent with this role, Notch1 downregulation in Lrig1^+^ endothelial cells may disrupt their resting state and activate a pro‐adhesion phenotype, especially in the context of NAPH‐induced injury and inflammation. This change could enhance the recruitment of circulating monocytes and leukocytes and facilitate their transendothelial migration into the VF damaged region. In homozygous mutants, these alterations persisted beyond the typical healing period [3, 4], which is generally completed within 1 week post‐injury, highlighting an additional role for Notch1 in the coordination of the timing of these repair‐associated processes.

Multiple injury models have been used to investigate VF mucosal regeneration [25, 26, 27]. Mechanical approaches, including abrasion or stripping, are widely used to induce VF mucosal damage involving the epithelium and lamina propria. In the abrasion model, repeated wire brush rubbing damages the VF mucosa and extends to the superficial thyroarytenoid muscle by day 1, followed by increased epithelial thickness on day 3 and 5, whereas increased proliferative and basal cells on day 3 [28]. In the stripping model, the epithelium and mucosa are removed using a gauge needle and microforceps. Partial epithelial coverage occurs by day 1, complete re‐epithelization by day 3, and reduced cell numbers with partial squamous‐like coverage by day 7 [29]. These studies indicate that epithelial coverage is largely restored within 3–5 days after mechanical VF injury in mice. Although clinically relevant, these models may produce variable lesion depths and disrupt both epithelium and underlying lamina propria. Consequently, the repair process may be affected by stromal injury, extracellular matrix remodeling, and procedure‐dependent variability [4, 26].

In contrast, NAPH induces chemically mediated epithelial injury by selectively ablating specific epithelial cell populations in the airway and larynx/VF [17, 21, 30]. Intraperitoneal injection avoids direct surgical disruption of the VF lamina propria, reduces procedure‐dependent variability, and provides a reproducible repair timeline, making this model suitable for mechanistic studies of epithelial repair. NAPH is also biologically and clinically relevant as a common environmental toxicant associated with adverse respiratory health outcomes [31]. Accordingly, it has been widely used to investigate airway epithelial injury and regeneration. In bronchiolar epithelium, NAPH causes rapid shifts in epithelial cell composition within 48 h, including non‐ciliated cells' exfoliation and transformation of ciliated cells into a squamous cell population [32]. In VF epithelium, NAPH‐induced epithelial damage is evident by day 1, followed by epithelial hyperplasia and increased squamous stratification by day 3; hyperplastic changes were largely resolved by 1 week [21, 33]. Using this model, we found that Notch1 deletion in quiescent Lrig1^+^ cells intensified repair‐associated epithelial changes and resulted in only partial recovery by 1 week post‐injury, especially in homozygous animals, suggesting a delayed healing response.

One of the key events during VF healing is transient epithelial hyperplasia, which is driven by intense basal cell proliferation to rapidly replace lost cells and restore the epithelium, typically peaking at 3 days post‐injury [9]. Our findings demonstrate that Notch1 downregulation keeps basal cells in a proliferative state even after 3 days post injury, which may delay the hyperplasia resolution. If not resolved in a timely manner, persistent hyperplasia can lead to epithelial thickening and may negatively impact VF vibratory function [34, 35, 36].

The next event in the healing process is keratin deposition, which strengthens the epithelium and increases its resistance to mechanical stress and further damage. KRT14, a keratin associated with stratified squamous epithelia, is primarily expressed in basal cell compartments [9]. Previously, lineage‐tracing studies revealed that NAPH injury induces KRT14 upregulation, peaking between 3 and 6 days, and KRT14^+^ basal cell can give rise to Clara‐like and ciliated cells in airway epithelium [37]. In VF epithelium, KRT14 expression expands throughout the epithelium on day 3 and 5 post‐injury [9]. In the present study, Notch1 deletion caused marked expansion of KRT14 expression toward the laryngeal squamo‐respiratory junction, a transition zone between stratified squamous and pseudostratified muco‐ciliary epithelium with poorly differentiated cells [38]. KRT13 is typically expressed in the luminal layer of stratified squamous epithelium and serves as a marker of epithelial differentiation [39]. In our study, KRT13 was strongly expressed throughout suprabasal layers and remained elevated up to 1 week post‐injury after Notch1 deletion. KRT17 is a type I acidic keratin that is typically induced under conditions of epithelial stress, inflammation, and wound repair [40, 41]. It is ectopically expressed in inflammatory skin wounds and epithelial carcinomas [42, 43], and elevated KRT17 expression has also been reported in oropharyngeal squamous cell carcinoma [44, 45]. In the present study, KRT17 upregulation in Notch1 deficient mice was sustained at 1 week post‐injury but remained restricted on the VF epithelium. Collectively, these findings suggest that Notch1 loss enhanced and prolonged keratin deposition during VF re‐epithelialization. Persistent keratin accumulation may alter epithelial immune cell responses and barrier function as well as contribute to hyperkeratinization, thereby stiffening the epithelial layer and potentially impairing VF vibration [46, 47].

Lastly, inflammation is essential for initiating and regulating wound healing [48]. Early inflammation promotes immune cells recruitment, with neutrophil infiltration typically peaking at 24 h and macrophage infiltration at 48 h after injury [4]. Controlled inflammation supports proper wound healing and scar reduction [48, 49] In contrast, persistent or chronic inflammation may promote pathological VF mucosa remodeling, leading to more severe and long‐lasting VF lesions [50, 51]. Besides the role of endothelial cells in immune cell infiltration, altered epithelial‐immune crosstalk may also contribute to sustained inflammation during VF repair. One possible mechanism is that Notch1‐deficient epithelial cells adopt a stress‐associated or transitional epithelial state, as reflected by increased KRT13/KRT17 expression. This state may alter the production of inflammatory or chemotactic signals thereby driving macrophage recruitment. In a pulmonary fibrosis study, transitional epithelial cells marked by keratin 8 were shown to modulate chemokine expression and promote macrophage accumulation, which may further reinforce epithelial remodeling and fibroblast activation [52]. Although chemokine expression and fibroblast activation was not directly examined in this study, our findings raise the possibility that Notch1 loss may sustain a persistent epithelial‐macrophage inflammatory loop that delays resolution of VF epithelial repair.

Several limitations should be noted. First, although Notch1 loss significantly disrupts VF re‐epithelialization, the downstream molecular mechanisms by which Notch1 regulates basal cell expansion, differentiation, and inflammation remain incompletely defined. In particular, Notch1‐dependent transcriptional programs and signaling pathways that coordinate epithelial regeneration warrant further investigation. Second, our assessment of inflammation was limited to macrophage presence based on IBA1 staining, without evaluation of macrophage activation states, cytokine production, or broader inflammatory signaling. Therefore, the functional contribution of immune responses to the observed epithelial dysregulation remains to be fully elucidated. Future studies incorporating detailed immune profiling and functional assays will be important to better define epithelial–immune interactions during VF repair.

## 5 Conclusion

In conclusion, our study demonstrates that Notch1 signaling regulates epithelial responses and repair timing in VF epithelium following acute injury, particularly within quiescent Lrig1^+^ cell populations. Notch1 loss activates VF basal epithelial cells and sustains them in a proliferative state, while suprabasal and luminal cells exhibit increased keratin deposition, accompanied by elevated immune cell infiltration. These repair‐associated processes persist beyond the typical healing period, highlighting Notch1's essential role in coordinating both structural and functional aspects of epithelial regeneration.

These findings provide valuable insights into stem cells' behavior during VF injury and highlight Notch1 as a key regulator in epithelial homeostasis and repair, with potential implications for studying VF diseases.

## Funding

This work was supported by the National Institutes of Health, R01DC004336, R01DC020734.

## Conflicts of Interest

The authors declare no conflicts of interest.

## Supporting information


**Figure S1:** Agarose gel electrophoresis (2%) showing the (A) Lrig1Cre^ERT2/+^, (B) Lrig1Cre^ERT2/+^Notch1^F/+^, (C) Lrig1Cre^ERT2/+^Notch1^F/F^. Lrig1Cre^ERT2/+^(100 bp), Notch1 Mutant(281 bp), Notch1 Wild type(231 bp).
**Table S1:** List of primers used.
**Table S2:** List of primary and secondary antibodies used.

## Data Availability

The data that support the findings of this study are available from the corresponding author upon reasonable request.
