# Supplementary material for: Effect of Notch1 on Vocal Fold Re‐Epithelization After Acute Injury
Source: Laryngoscope. 2026 Jul 15;136(12):5434–43. doi: 10.1002/lary.70760 (PMC13765423; doi:10.1002/lary.70760)
Supplement: Supplementary file 1 [file LARY-136-5434-s001.docx]

**Supplementary Materials**

**Genotyping Analysis**

Genomic DNA was extracted from mouse ear punches using an alkaline lysis method. A single ear punch was placed into a microcentrifuge tube containing 200 μl of Solution A (1.25 ml 10 M NaOH + 200 µl 0.5 M EDTA, bring to 500 ml with water). Samples were incubated in 95°C water bath for 30 minutes, followed by the addiction of 200 ul Solution B (40 ml 0.5 M Tris pH 8, bring to 500 ml with water). The samples were vortexed briefly and centrifuged at 13,000 rpm for 5 minutes. The genomic DNA were collated and stored at 4°C until use.

The genotyping was performed by polymerase chain reaction (PCR) following the protocol recommended by the Jackson Laboratory. Representative genotyping results are shown in Supplementary Figure1.The primers sequence was show in the Supplementary Table1.

**Supplementary Figure S1.** Agarose gel electrophoresis (2%) showing the A. Lrig1Cre^ERT2/+^, B. Lrig1Cre^ERT2/+^Notch1^F/+^, C. Lrig1Cre^ERT2/+^Notch1^F/F^. Lrig1Cre^ERT2/+^(100bp), Notch1 Mutant(281bp), Notch1 Wild type(231bp)

| **Gene** | **Forward sequence 5’→ 3’** | **Reverse sequence 5’→ 3’** |
| --- | --- | --- |
| Lrig1 | GCG GTC TGG CAG TAA AAA CTA TC | GTG AAA CAG CAT TGC TGT CAC TT |
| Notch1 | TGC CCT TTC CTT AAA AGT GG | GCC TAC TCC GAC ACC CAA TA |

**Supplementary Table S1****.** List of primers used

| **Antibody** | **Species** | **Dilution** | **Cat#** | **Vendor** |
| --- | --- | --- | --- | --- |
| P63 | Mouse | 1:100 | CM163A | Biocare Medical |
| Ki-67 | Rabbit | 1:150 | Ab16667 | Abcam |
| KRT14 | Rabbit | 1:150 | 10143-1-AP | Proteintech |
| KRT13 | Mouse | 1:100 | MA1-35542 | Invitrogen |
| KRT17 | Rabbit | 1:50 | Ab109725 | Abcam |
| IBA1 | Rabbit | 1:100 | Ab178847 | Abcam |
| Alexa Fluor 488 | Goat | 1:500 | A20000 | ThermoFisher |
| Cy3 | Goat | 1:300 | AB_2338680 | Jackson ImmunoResearch |

**Supplementary Table S2****.** List of primary and secondary antibodies used
